# Expression of in vivo biotinylated recombinant antigens SAG1 and SAG2A from *Toxoplasma gondii* for improved seroepidemiological bead-based multiplex assays

**DOI:** 10.1186/s12896-020-00646-7

**Published:** 2020-10-06

**Authors:** Sandra Klein, Daniel Stern, Frank Seeber

**Affiliations:** 1 FG 16 − Mycotic and Parasitic Agents and Mycobacteria, Robert Koch Institute, 13353 Berlin, Germany; 2 ZBS 3 − Biological Toxins, Robert Koch Institute, 13353 Berlin, Germany

**Keywords:** *Toxoplasma gondii*, Surface antigens, Bead-based multiplex assay, Biotinylation tag, Diagnosis, Seroepidemiology

## Abstract

**Background:**

Few bead-based multiplex assays have been described that detect antibodies against the protozoan parasite *Toxoplasma gondii* in large-scale seroepidemiological surveys. Moreover, each multiplex assay has specific variations or limitations, such as the use of truncated or fusion proteins as antigens, potentially masking important epitopes. Consequently, such an assay must be developed by interested groups as none is commercially available.

**Results:**

We report the bacterial expression and use of N-terminal fusion-free, soluble, in vivo biotinylated recombinant surface antigens SAG1 and SAG2A for the detection of anti-*T. gondii* IgG antibodies. The expression system relies on three compatible plasmids. An expression construct produces a fusion of maltose-binding protein with SAG1 (or SAG2A), separated by a TEV protease cleavage site, followed by a peptide sequence recognized by *E. coli* biotin ligase BirA (AviTag), and a terminal six histidine tag for affinity purification. TEV protease and BirA are encoded on a second plasmid, and their expression leads to proteolytic cleavage of the fusion protein and a single biotinylated lysine within the AviTag by BirA. Correct folding of the parasite proteins is dependent on proper disulfide bonding, which is facilitated by a sulfhydryl oxidase and a protein disulfide isomerase, encoded on the third plasmid. The C-terminal biotinylation allowed the oriented, reproducible coupling of the purified surface antigens to magnetic Luminex beads, requiring only minute amounts of protein per determination. We showed that an N-terminal fusion partner such as maltose-binding protein negatively influenced antibody binding, confirming that access to SAG1’s N-terminal epitopes is important for antibody recognition. We validated our bead-based multiplex assay with human sera previously tested with commercial diagnostic assays and found concordance of 98–100% regarding both, sensitivity and specificity, even when only biotinylated SAG1 was used as antigen.

**Conclusions:**

Our recombinant in vivo-biotinylated *T. gondii* antigens offer distinct advantages compared to previously described proteins used in multiplex serological assays for *T. gondii*. They offer a cheap, specific and sensitive alternative to either parasite lysates or eukaryotic-cell expressed SAG1/SAG2A for BBMA and other formats. The described general expression strategy can also be used for other antigens where oriented immobilization is key for sensitive recognition by antibodies and ligands.

## Background

Toxoplasmosis is caused by the zoonotic protozoan parasite *Toxoplasma gondii*, a relative of *Plasmodium* spp., the malaria-causing pathogen. While the acute infection of healthy subjects with *T. gondii* is usually mild, an infection of severely immunocompromised individuals or of fetuses from seronegative pregnant women can have serious medical consequences, potentially leading to death if not treated [1].

Infection occurs either through the ingestion of undercooked or poorly processed meat from infected animals or via uptake of water or food contaminated by the environmentally resistant form shed by infected cats into the environment. Toxoplasmosis is amongst the most prevalent infectious diseases worldwide and it is estimated that roughly one third of the global human population is chronically infected [1, 2]. However, seroprevalence varies considerably both within and between countries [3], and it is thought to be dependent on various environmental factors including eating habits, food preferences, and contact with cats. Establishing statistically sound correlations between such risk factors and seropositivity requires that large representative cohorts are tested for antibodies directed against *T. gondii*. For example, the most reported seroprevalence data come from women, being either in child-bearing age or pregnant [3, 4]. This shortfall highlights the need for more representative large-scale studies; however, these comprehensive seroprevalence studies are often hampered by suboptimal tests. Thus, integrated surveillance approaches for public health that include multiplex serological assays for many pathogen antigens are needed [5–7]. Consequently, the establishment and optimization of bead-based multiplex assays (BBMA) based on the xMAP technology [8] that include *T. gondii* antigens are of considerable interest [5].

While a few studies have reported the application of bead-based multiplex assays that include *T. gondii* antigens, such assays differ substantially in detail [9–13] and none are commercially available. Given our interest in the epidemiology of *T. gondii* [14], we developed a bead-based multiplex assay based on the recombinant *T. gondii* antigens SAG1 (SRS29B) and SAG2A (SRS34A) – two of the most widely used diagnostic antigens [15]. Both are immunodominant surface proteins and elicit a strong humoral immune response in humans and infected animals [16]. However, recombinant SAG1 can be problematic to express in *E. coli*, and also may not mimic the native protein conformation. This difficulty is due to the six disulfide bonds required for proper conformation [17] that strongly influence immune recognition by human sera infected with *T. gondii* [18]. SAG1 has therefore been expressed in various eukaryotic hosts [19–23], yet this is more expensive and time-consuming. We here describe an optimized bacterial expression system that ensured correctly folded, soluble protein with oriented attachment via biotin binding to streptavidin-coated magnetic beads, which provided optimal presentation and antigenicity.

## Results

### Expression strategy of recombinant SAG1 (SAG2A)

**Fig. 1:**
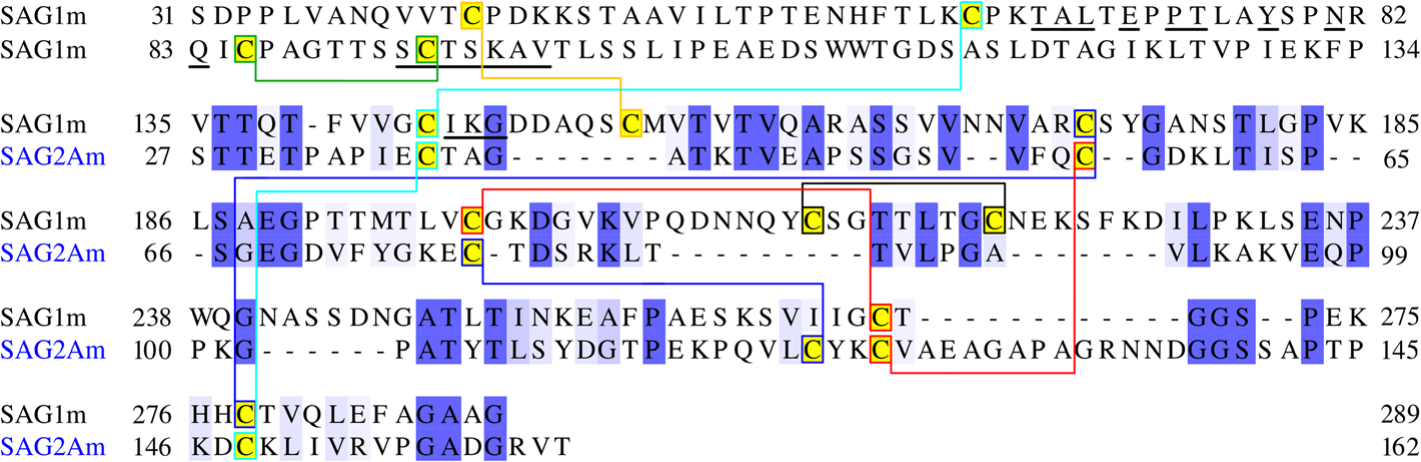
Sequence comparison between the mature forms of SAG1 and SAG2A. The residue numbering is according to the full-length, unprocessed proteins, whereas only the sequences without N- and C-terminal topogenic sequences are displayed. The sequence identity is 24.3% and similarity 34.1%, respectively. All cysteines are highlighted in yellow. Matching colors of the boxes in each sequence indicate the residues involved in the respective disulfide bond and are connected by lines. The three matching Cys pairs of SAG2A were inferred from [17]. The amino acids in each monomer of SAG1 forming the epitope are underlined according to [30]. It consists of Thr_67_-Ala_68_-Leu_69_, Glu_71_, Pro_73_-Thr_74_, Tyr_77_, Asn_80_, Gln_82_ and Ser_91_-Cys_92_-Thr_93_-Ser_94_-Lys_95_-Ala_96_-Val_97_, all part of a loop. In a second much shorter loop of the structure there are three consecutive residues, Ile_144_-Lys_145_-Gly_146_, that are part of the epitope. No data for SAG2A exists in this respect

The GPI-anchored surface proteins of *T. gondii* tachyzoites, which include SAG1 and SAG2A, have well-known N- and C-terminal topogenic signal sequences [16, 24]. However, surprisingly little attention has been paid in the past to their potential influence on antigenicity of the recombinant proteins used for diagnostic purposes when the N- and C-terminal signal sequences are left intact (see e.g. [11, 25–29]). Additionally, deletions or N-terminal fusions with relatively large glutathione-S-transferase (GST) protein tags have both been used, which may impact antigenicity. However, in the case of dimeric SAG1, a previous study by Graille et al. [30] provided convincing evidence that a conformational epitope of the monomers, important for recognition by human antibodies from infected individuals, is found at the N-terminus of the mature protein (Fig. 1).

**Fig. 2:**
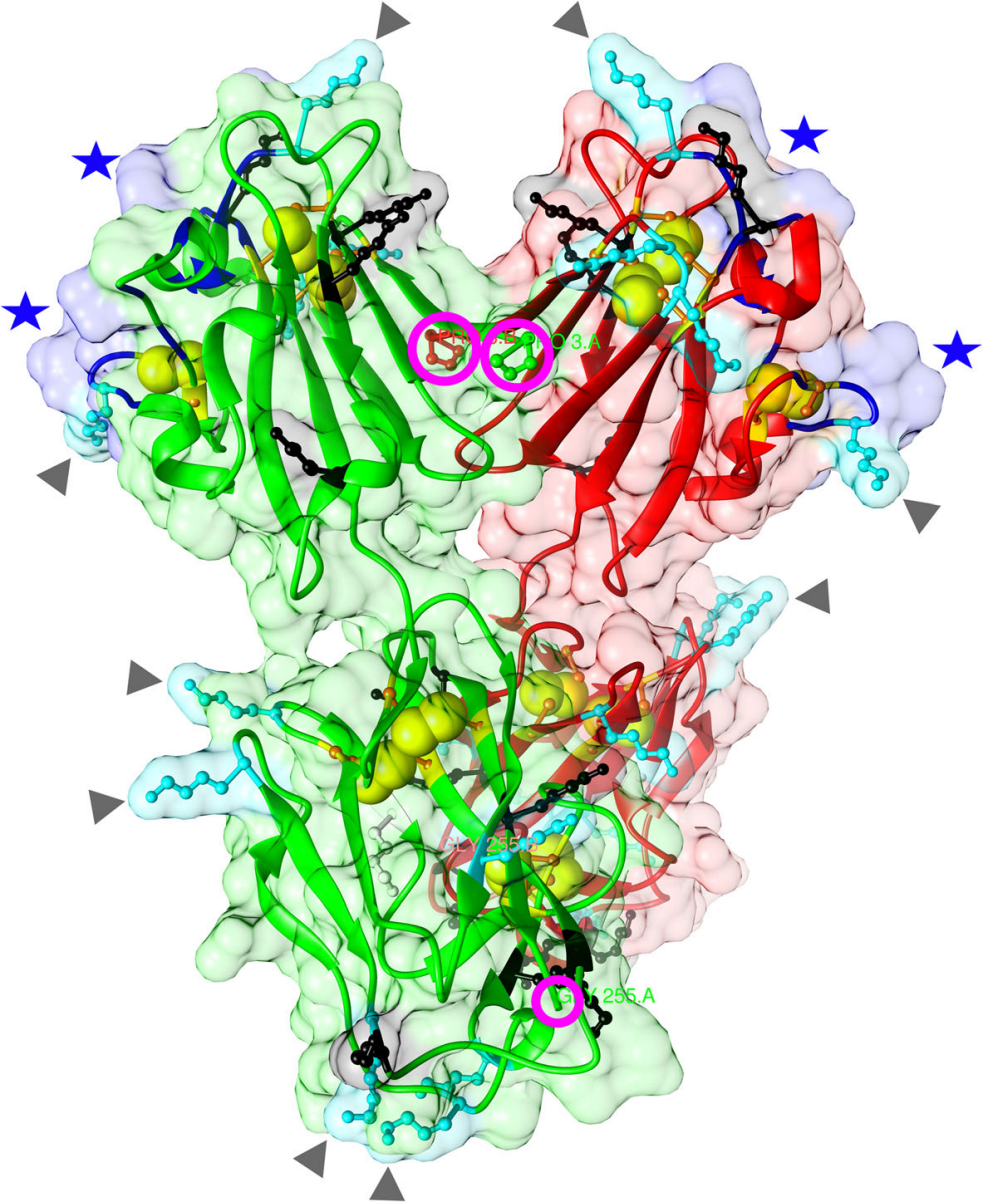
3D structure of the SAG1 dimer. This image is based on PDB 1ynt [17]. The six disulfide bonds in each monomer are depicted as yellow “double balls”. The two magenta circles at the top mark the N-terminal proline of the solved structure (Pro_34_ in Fig. 1); the single smaller one the C-terminal glycine (Gly_286_ in Fig. 1) in one of the monomers. Ball-and-stick structures in black and cyan are lysines, whereby the grey arrow heads mark those that are particularly surface-exposed (also visible by the cyan-colored surface cloud). The discontinuous epitope of each monomer is apparent by its blue surface cloud (blue stars) and the individual deep blue-colored residues (see Fig. 1 for their position)

This conclusion was based on the 3D structure of a complex of a monoclonal antibody (mAb) bound to SAG1. This mAb competes very efficiently with the binding of human antibodies by making contact with discontinuous N-terminal residues, forming what appears to be the immunodominant epitope of SAG1 (highlighted in blue in the dimeric form; Fig. 2) [30]. Thus, we considered it to be important to conserve the structural integrity, in particular access to the N-terminus of the protein, when expressing recombinant SAG1. Consequently, full length, non-fused and correctly folded dimeric SAG1 [17] is considered to be the best antigen for optimal recognition by human antibodies.

Furthermore, since our main objective was to use SAG1 in BBMA where the usual immobilization of proteins to the Luminex microbeads is via chemical coupling we reasoned that this could affect SAG1’s recognition by antibodies. In this immobilization procedure lysine side chains, in particular those that are surface-exposed, are coupled in a non-selective manner via EDC (1-Ethyl-3-[3- dimethylaminopropyl]carbodiimide hydrochloride) and Sulfo-NHS (N-hydroxysulfosuccinimide) to the carboxy groups of the beads. Dimeric SAG1 contains 40 lysine residues, of which 38 have a calculated solvent accessible surface area, SAS, (as determined in the known 3D structure) ≥ 40 Å^2^ (highlighted in black in Fig. 2). Of those, 21 have a SAS ≥ 100 Å^2^ (cyan in Fig. 2), providing a rich landscape of potential attachment sites. Several lie within or very close to the dominant epitope, thereby possibly destroying or severely affecting antibody binding. Consequently, a targeted immobilization strategy that would allow SAG1 to be coupled exclusively via its C-terminal end (similar to its GPI anchor attachment in the plasma membrane [24, 30];) could improve immune recognition by human sera.

It is long known that efficient humoral SAG1 recognition depends on correct folding of the protein. From Fig. 2 it is apparent that proper formation of three of the six disulfide bonds of SAG1 will directly affect the formation of the dominant epitope (see also Additional file 1: Movie S1). Recombinant truncated SAG1 versions lacking any of these disulfide bonds (e.g. [31]) will therefore be suboptimal.

Taken all this into account our rationale for the expression construct for SAG1 (and also SAG2A) was as follows: the recombinant protein should

- contain the entire mature coding region to include all possible epitopes of the native protein (Fig. 1),
- allow correct S-S bonding, thereby maximizing correct folding,
- allow oriented, controllable immobilization on magnetic beads [32],
- possess a cleavable fusion partner to aid in increased solubility.

### Construction of a three-plasmid expression system for SAG1/SAG2A

**Fig. 3:**
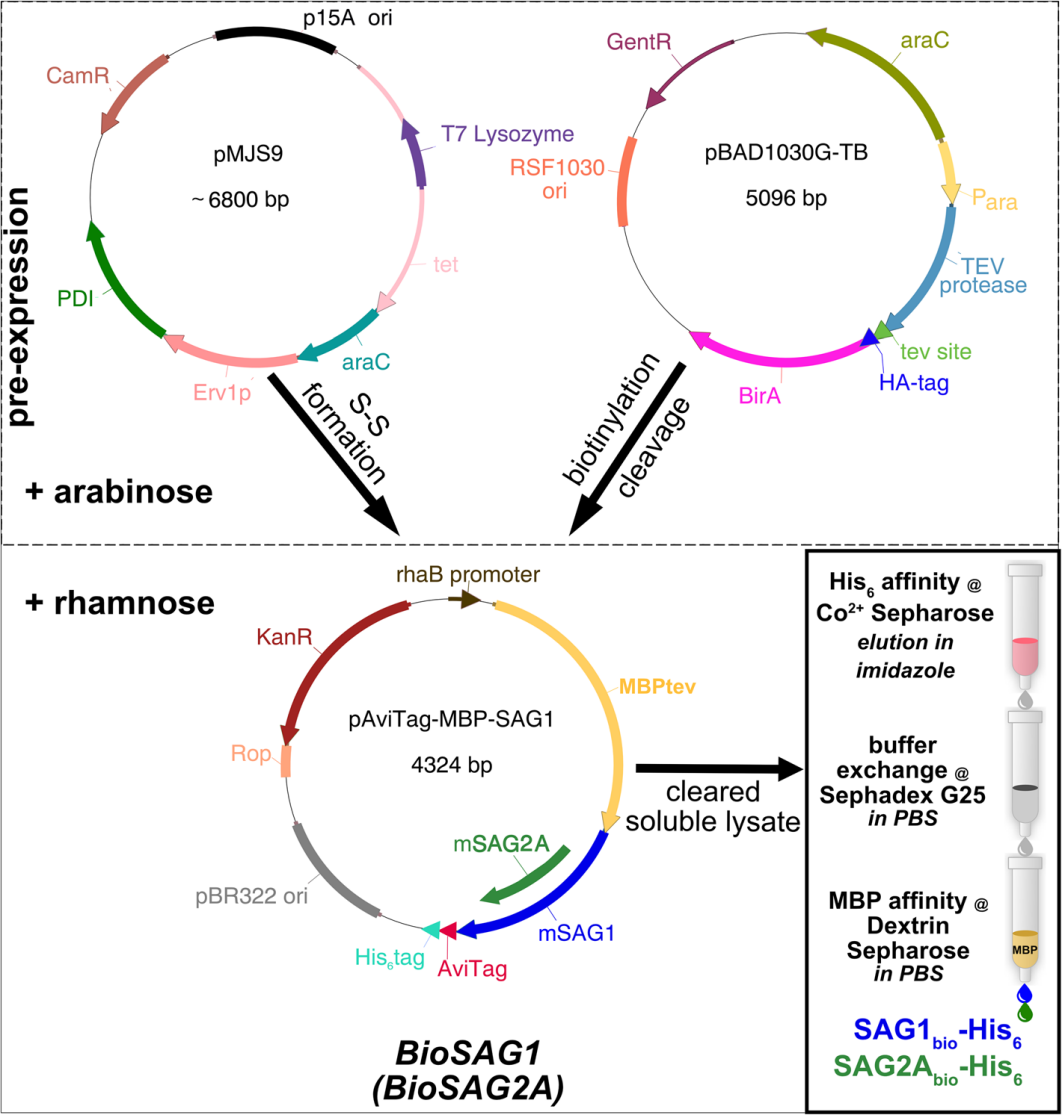
Expression and purification scheme of SAG1_bio_(SAG2A_bio_)-His_6_. Protein expression of pMJS9 and pBAD1030G-TB is initiated by addition of arabinose (pre-expression), followed after 30 min by rhamnose. The produced MBP_tev_-SAG1-AviTag-His_6_ is subsequently biotinylated and the fusion protein is cleaved by TEV. The cleared *BioSAG1* (*BioSAG2A*) lysate is purified by a three-step procedure – affinity chromatography, buffer exchange and removal of MPB-containing proteins

To accomplish the above aims, pAviTag-MBP-SAG1 and pAviTag-MBP-SAG2A expression plasmids were designed (Fig. 3; Additional file 2 and 3: Sequence S1 and S2) and assembled (Methods). Both SAG1 and SAG2A were N-terminally fused with maltose binding protein (MBP), which promotes enhanced solubility during translation and folding [33]. After expression, MBP is later cleaved in situ so that access of antibody to the epitopes is not inhibited, as discussed above. Therefore, the constructs included MBP followed by a cleavage recognition site (tev) for the Tobacco Etch Virus (TEV) protease [34] that for SAG1 results in mature protein with Ser_31_ as the most N-terminal amino acid (see Additional file 4: Supplementary Figure S1A). The putative GPI-attachment site (Gly_289_) at the C-terminus was followed by a 4 kDa peptide sequence (AviTag) that can be recognized by *E. coli* biotin ligase BirA, which catalyzes the attachment of biotin at the lysine within the sequence [35]. The resulting biotinylated protein can thus be immobilized and oriented via its C-terminal end by biotin-streptavidin interaction. The AviTag was followed by a His_6_ tag for affinity purification by metal chelate affinity chromatography (Fig. 3; Additional file 4: Supplementary Fig. S1A).

The six disulfide bonds of SAG1 pose a challenge for correct folding in a reducing environment like the cytosol of *E. coli* [23]. We therefore chose the system developed by Nguyen et al. [36] that allows for improved cytoplasmic disulfide bond formation in *E. coli* (called ‘CyDisCo’). This consists of the pre-expression of a sulfhydryl oxidase combined with a protein disulfide isomerase (PDI) and can be transformed into an *E. coli* strain with gor and trxB gene deletions [36], which are involved in disulfide bond reduction. Their deletion and the additional expression of DsbC in the bacterial cytoplasm results in better disulfide bond formation in the *E. coli* strain SHuffle [37]. The plasmid pMJS9 also contained genes for codon-optimized sulfhydryl oxidase Erv1p from *Saccharomyces cerevisiae* and codon-optimized human PDI, which is regulated by an arabinose-inducible promoter [36] (Fig. 3).

As BirA is present only in very small amounts in *E. coli* cells, BirA overexpression is required for substantial in vivo biotinylation [38]. Thus, we also used a third plasmid, pBAD1030G-TB, which expresses TEV protease and BirA (Fig. 3; Additional file 4: Supplementary Figure S1B; Additional file 5: Sequence S3). Although BirA has been shown to be active as an N-terminal fusion protein [39] we opted for a construct where the sequences for TEV protease and BirA are separated by a tev cleavage site. Such an arrangement results in post-translational self-processing of the fusion protein in stoichiometric amounts of the individual protein entities [40].

The *E. coli* SHuffle strain transformed with the three plasmids (each possessing a different resistance gene as well as compatible replication origins) was named *BioSAG1* (Fig. 3). A strain with pAviTag-MBP-SAG2A was similarly constructed and termed *BioSAG2A*.

### Expression, purification and characterization of biotinylated SAG1 and SAG2A

**Fig. 4:**
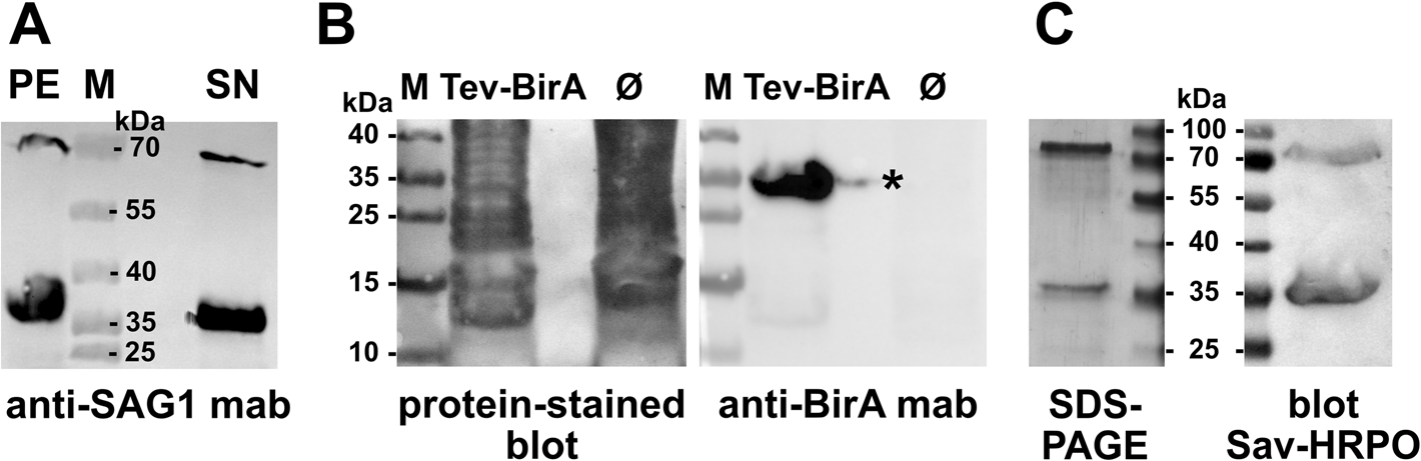
Production of soluble fusion-free SAG1 and self-processing of TEV_tev_-BirA fusion protein. **a** Western blot of insoluble (pellet) and soluble fractions (SN) of an induced *BioSAG1* lysate with anti-SAG1 mab DG52, indicating substantial soluble and processed protein production of SAG1. **b** Stained membrane of a bacterial lysate with (TEV-BirA) or without (Ø) plasmid pBAD1030G-TB (left) followed by detection of BirA by a mouse mab directed against it (right). * contamination from left lane. **c** Silver-stained SDS-PAG of purified SAG1_bio_-His_6_ (left) containing uncleaved MBP_tev_-SAG1_bio_-His_6_ and detection of biotinylation by Sav-HRPO (right). Images of gels and blots were cropped; full-length blots/gels are presented in Additional file 8: Supplementary Fig. S2

Recombinant protein production in the *BioSAG* strains is initiated by the addition of arabinose, which induces expression of Erv1p and PDI on pMJS9 as well as TEV protease and BirA on pBAD1030G-TB due to the presence of the arabinose-inducible promoter on both plasmids. Such pre-expression has been reported previously to increase correct S-S bond formation [36] as well as biotinylation [38]. Next, rhamnose is added to produce MBP_tev_-SAG1-AviTag-His_6_ (MBP_tev_-SAG2A-AviTag-His_6_), on which the pre-expressed proteins act upon (i.e., forming disulfide bridges and proper folding by Erv1p, PDI and DsbC; cleavage of MBP_tev_-SAG1 and TEV_tev_-BirA by TEV protease; biotinylation by BirA). This regimen results in the soluble expression of an N-terminal fusion-free SAG1_bio_-His_6_. As shown in Fig. 4a, the cell lysate of *BioSAG1* was separated into soluble and insoluble fractions, which were subsequently analyzed by SDS-PAGE and immunoblotting with the mouse mab (DG52) that recognizes a disulfide bond-dependent conformational epitope [18, 23, 41]. While the pellet still contained substantial amounts of insoluble protein, DG52 recognizes its epitope in both fractions, which is indicative of proper disulfide bond generation. The in situ cleavage of MBP by TEV protease was rather efficient since only small amounts of DG52 reactivity was seen at a size of > 70 kDa, which is the size of the fusion protein (calculated M_w_ of 74,8 kDa). As shown in Fig. 4b, BirA was detected as a single protein band of the expected size (~ 37 kDa) upon induction only in a strain that contains pBAD1030G-TB. This indicated successful self-cleavage of the TEV_tev_-BirA fusion protein. The endogenous BirA was undetectable in a strain lacking the plasmid, which is consistent with the low endogenous amount of the BirA ligase under standard growth conditions [42, 43].

**Fig. 5:**
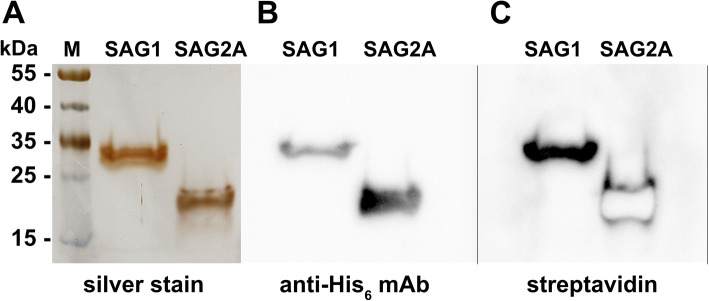
Purity and biotinylation assessment of SAG1_bio_-His_6_ and SAG2A_bio_-His_6_. **a** Silver staining of SDS-PAG of purified proteins. **b** Western blot analysis (same protein amounts as in A) with anti-His_6_ antibody to detect the proteins, or **c**, streptavidin, both coupled to horseradish peroxidase. The “bleached” signal for SAG2A_bio_-His_6_ in C was due to very strong chemiluminescence. Images of gels and blots were cropped; full-length blots/gels are presented in Additional file 8: Supplementary Fig. S2

The addition of a second affinity chromatography step to the purification procedure (see Fig. 3 and Methods) allowed for the entire MBP (cleaved or as fusion) to be retained on the dextrin affinity column after prior buffer exchange of the eluate on a desalting column. This led to the purification of SAG1_bio_-His_6_ and SAG2A_bio_-His_6_ to near homogeneity (Fig. 5a). Both proteins were also biotinylated (Fig. 5b, c), as indicated by probing the blot with Sav.

Using this expression system, we could purify several hundred micrograms of pure SAG1_bio_-His_6_ and SAG2A_bio_-His_6,_ respectively, from one liter of bacterial culture. It should be noted, however, that protein preparations that contain uncleaved MBP_tev_-SAG1_bio_-His_6_ (Fig. 4c) that had been co-purified on the metal chelate affinity column could still be used efficiently for BBMA, with a higher overall yield than the optimized 3-step protocol.

### Bead-based multiplex assay with biotinylated SAG1 and SAG2A as antigens

The overall aim of this study was to establish a BBMA with biotinylated SAG1 and SAG2A as antigens for analyzing seroconversion resulting from *T. gondii* infection in humans. Magnetic beads have distinct advantages over non-magnetic beads, including the ease of processing and higher bead recovery [8, 44]. Since at the beginning of these studies the Sav-coated MagPlex® microbeads were not commercially available, we custom-prepared them by chemical coupling of Sav to various bead regions (see Methods).

**Fig. 6:**
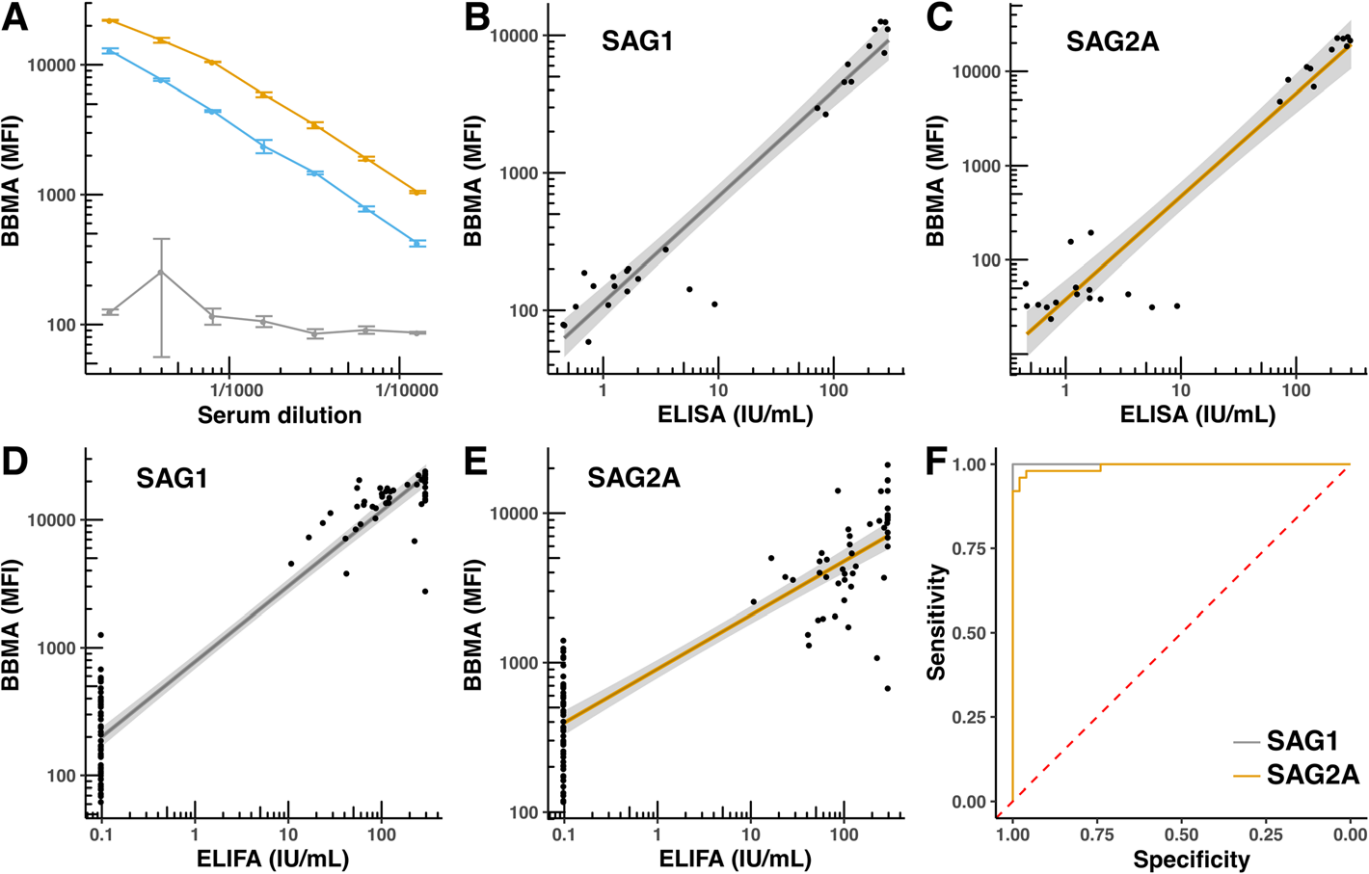
Evaluation of anti-SAG1_bio_-His_6_ and anti-SAG2A_bio_-His_6_ responses by BBMA. **a** Titration of human sera with different anti-*T. gondii* titers (in IU) against SAG1_bio_-His_6_ (10 ng/1500 Sav-coated beads per sample): Orange - highly positive (> 200 IU/mL), blue - medium positive (63 IU/mL) and gray - negative serum, respectively. **b**, **c** Comparison of BBMA MFI of 11 anti-*T. gondii* antibody-positive and 16 *-*negative sera with titers determined by a commercial ELISA (Euroimmun) for SAG1_bio_-His_6_ (**b**) and SAG2A_bio_-His_6_ (**c**). Pearson’s correlation coefficients: 0.96 for SAG1_bio_-His_6_ and 0.94 for SAG2A_bio_-His_6_. **d**, **e** Comparison of BBMA MFI of 50 positive and 50 negative sera with titers determined by a commercial ELIFA for SAG1_bio_-His_6_ (**d**) and SAG2A_bio_-His_6_ (**e**). Pearson’s correlation coefficients: 0.96 for SAG1_bio_-His_6_ and 0.89 for SAG2A_bio_-His_6_. Shaded areas in **b**-**e** indicate 95% CI. **f** Receiver-operator curve comparing the BBMA with the commercial ELIFA for SAG1_bio_-His_6_ (gray line) and SAG2A_bio_-His_6_ (orange line). Area under curve: 1.0 for SAG1_bio_-His_6_ and 0.99 for SAG2A_bio_-His_6_

We determined the minimal amount of protein that would be required to obtain maximal MFI with human control sera of known anti-*T*. *gondii* IgG antibody titers (Fig. 6a). Ten nanograms per serum sample of a SAG1_bio_-His_6_ preparation similar to Fig. 4c were sufficient to obtain an MFI of > 25,000, the maximum MFI value that is usually informative. The human control sera could be titrated down to more than a 1:12,000 dilution and still possess positive signals above those obtained by a negative control serum (Fig. 6a). This indicates that the obtained dose-response curve also allows low amounts of antibodies to be specifically detected.

Using a panel of 27 human sera that were previously tested positive (11 sera) or negative (16 sera) for anti-*T. gondii* antibodies using a commercial ELISA (Euroimmun), both antigens allowed a clear distinction between those donors. We noted a good correlation between positive titers determined by the commercial test versus our BBMA titers (Fig. 6b and c). To verify these results, we analyzed an additional set of 50 positive and 50 negative sera (Fig. 6d and e). The titers in these human sera had been previously determined using a commercial, clinically-used automated ELIFA (bioMérieux). In comparative studies this commercial assay had shown a sensitivity above 99% and specificity above 98% [45]. We obtained similar results with SAG1_bio_-His_6_, which allowed for a perfect discrimination between positive and negative sera as classified by the ELIFA. In contrast, our analysis using SAG2A_bio_-His_6_ beads showed a slightly lower sensitivity and specificity of 98% each (see also Table 1).

**Table 1:** Comparison of published BBMAs for detection of anti-*T. gondii* IgG antibodies

antigen (final source)	aa	Coupling to beads (X/M)	# reference sera ^**1**^	specificity	sensitivity	antigen/beads	per 1 Mio beads [μg]	signal amplification via biotinylated ab?	Reference
SAG1_bio_-His_6_ (*E. coli*)	31–289	biotin-streptavidin (M)	27 / 102	1 / 1	1 / 1	10 μg/ 1.5 × 10^6^	6.7	no	this study
SAG2A_bio_-His_6_ (E. coli)	27–162	biotin- streptavidin (M)	27 / 102	1 / 0.98	1 / 0.98	10 μg/ 1.5 × 10^6^	6.7	no	this study
SAG1-Stag (E. coli)	61–300	chemical (M)	59	0.950	0.947	30 μg/ 1.25 × 10^6^	24	no	[29]
GST-SAG2A (E. coli)	27–173	chemical (X)	100	1	1	120 μg/12.5 × 10^6^	6	**yes**	[10]
cell lysate (*T. gondii*)	na	chemical (M)	20	1	1	na	na	no	[46]
cell lysate (*T. gondii*)	na	chemical (X)	80	1	1	na	na	**yes**	[13]
GST-SAG1 (HeLa cells)	ns	chemical (X)	5	1	1	5 μg/5 × 10^6^	1	**yes**	[12, 47]
GST-SAG1 (E. coli)	31–349 ^2^	GSH-casein affinity (X)	198	0.86	0.845	ns	ns	**yes**	[11]
GST-SAG2A (E. coli)	27–187	GSH-casein affinity (X)	198	0.86	0.926	ns	ns	**yes**	[11]

*M* MagPlex® or BioPlex® magnetic beads, *X* xMAP® non-magnetic beads, *ns* not specified; ^1^ sum of positive and negative sera; ^2^ SAG1 has only 336 aa

**Fig. 7:**
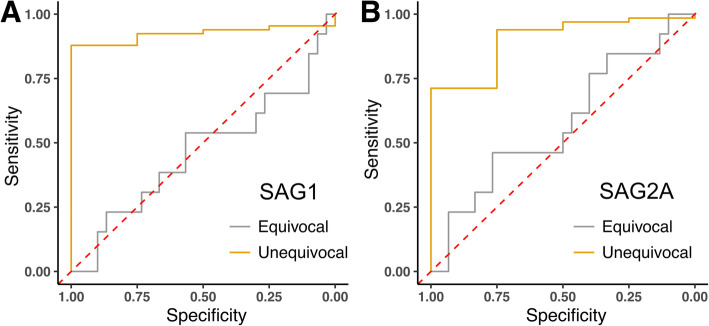
Discriminatory power between positive and negative sera around the ELIFA cut-off by SAG1_bio_-His_6_ and SAG2A_bio_-His_6_ employed in the BBMA. A total of 102 sera classified as negative (< 4 IU/mL), equivocal (4 to ≤8 IU/mL) or positive (≥ 8 IU/mL) by automated ELIFA were analyzed by BBMA. **a** Receiver-operator curve analysis with SAG1_bio_-His_6_ as antigen, or **b** SAG2A_bio_-His_6_. Area under curve for SAG1_bio_-His_6_: 0.47 for equivocal sera and 0.92 for unequivocal sera. Area under curve for SAG2A_bio_-His_6_: 0.58 for equivocal sera and 0.90 for unequivocal sera

Finally, the high diagnostic value of our recombinant proteins in a BBMA was indicated by our testing of a panel of 102 sera with titers slightly below or above the diagnostic cut-off of the ELIFA (8 IU/mL). In this assay, the sera between 4 and 8 IU/mL are classified as equivocal, while sera above 8 and below 4 IU/mL are considered positive or negative, respectively, by the manufacturer. By comparing these results with those values for our BBMA for SAG1_bio_-His_6_ and SAG2A_bio_-His_6_, the equivocal sera could also not be discriminated. This showed an almost perfect 50/50 ratio of positive and negative sera (Fig. 7). In contrast, using sera at ≥8 IU/mL or < 4 IU/mL, we were able to classify each with high confidence as either positive or negative. We conclude that a highly similar performance and sensitivity of our BBMA can be obtained as compared to that of commercial assays, even when sera close to the cut-off values are analyzed.

### N-terminal MBP influences binding of human antibodies to SAG1

**Fig. 8:**
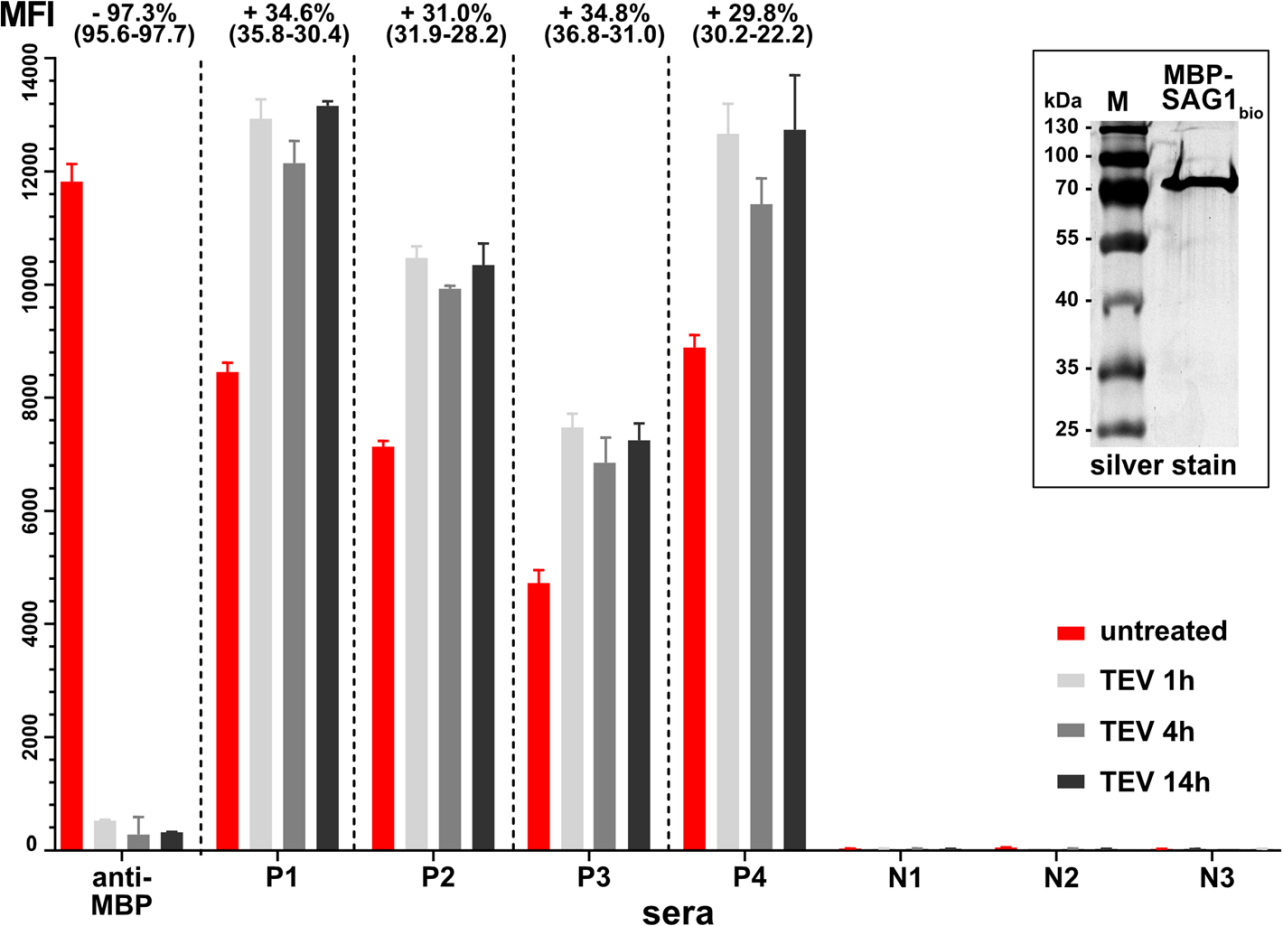
Recognition of SAG1_bio_-His_6_ in dependence of MBP as fusion partner. Inlet shows purified MBP_tev_-SAG1_bio_-His_6_ used in this assay_._ Red bars indicate a non-treated sample, whereas the grey/black bars represent samples that were treated for different time points with TEV protease. Percentages given compare mean MFI values of the respective three treated assays to those of the untreated controls (range in parentheses). P, positive human sera of differing titers; N, negative human sera. The gel image was cropped; the full-length gel is presented in Additional file 8: Supplementary Fig. S2

As a proof for our hypothesis that N-terminal fusions to SAG1 would influence the binding of human antibodies, we coupled MBP_tev_-SAG1_bio_-His_6_ purified from a strain devoid of TEV but expressing BirA (from pBAD1030G-B; Additional file 6: Sequence S4) to Sav-coated beads (Fig. 8 inlet). We then added TEV protease to one half of the beads to release MBP from SAG1 and incubated them for various time points. The cleavage was very efficient even after 1 h, which is indicated by only minute anti-MBP mab binding (Fig. 8). Since the amount of bead-bound SAG1_bio_ should be identical between both conditions, we probed these beads as well as TEV protease-untreated beads to quantitatively compare the binding of anti-SAG1-directed antibodies present in human sera. Whereas the negative sera showed no binding in any condition, the removal of MBP lead to a higher fluorescence intensity (30–35%; Fig. 8) with the four tested sera. The level of intensity was less pronounced (10–20%, depending on the serum) with lower amounts of initial protein (data not shown). Notably, the observed high activity of TEV protease on the fusion protein allowed the omission of both the in situ cleavage by plasmid-encoded TEV protease and the dextrin affinity column step. Instead, one could just rely on the in vitro cleavage protocol of MBP_tev_-SAG1_bio_-His_6_, purified only by metal chelate affinity chromatography.

We conclude that N-terminal fusion proteins do influence the binding of human antibodies to SAG1 and that their removal result in less protein being required for BBMA. However, uncleaved MBP_tev_-SAG1_bio_-His_6_ is still a very useful diagnostic antigen in this context.

## Discussion

We have described the production of biotinylated antigens SAG1 and SAG2A of *T. gondii* for BBMA applications that have distinct advantages as compared to those previously described in the literature (Table 1). Specifically, lower amounts of *E. coli*-derived SAG1_bio_-His_6_ are required per assay, even when using protein preparations containing proportions of uncleaved MBP_tev_-SAG1. Recombinant SAG1 produced in eukaryotic HeLa cells [12, 47] requires detection by a biotinylated secondary antibody, which is known to increase the sensitivity in BBMA [48] in order to reach the reported 1 μg/1 × 10^6^ beads. However, both of these components cause higher costs per determination. Even though our approach requires Sav as an additional compound, Sav can also be efficiently produced in *E. coli*, which further reduces costs [49, 50]. Using SAG2A_bio_ in BBMA resulted in higher MFI and thus an IU/mL with similar discriminatory power between seropositive and seronegative sera (Fig. 6), as was used successfully in prior studies [10, 11]. The specificity and sensitivity (accuracy) was in our hands slightly lower, however, as compared to SAG1_bio_. Combining both proteins into a single BBMA was shown recently to be essential for satisfactory accuracy [11]. In some humans with overall low anti-*T. gondii* antibody titers, the immune response might be directed toward more than one antigen, as has been reported for other *T. gondii* antigens [51]. Nevertheless, we show here that both proteins can be used alone with very high accuracy, even with challenging sera close to cut-off values of commercial assays. While we have not specifically tested sera derived from humans infected with different pathogens for cross-reactivity this can be evaluated in future large scale sero-epidemiological BBMA studies. However, SAG1 has been shown in the past to be a highly specific diagnostic antigen [15], able to discriminate infections caused even by closely related Apicomplexa [52, 53].

In another apicomplexan parasite, *Babesia* sp., GPI-anchored surface proteins and their soluble versions that are released after enzymatic cleavage can elicit antibodies of different parasite-neutralizing potency [54]. The authors suggested that different protein conformations (due to lost membrane anchorage) are responsible for this effect. For diagnostic purposes, we also considered it advantageous that SAG1 (SAG2A) should resemble the native protein on the parasite’s surface as much as possible. We mimicked the conditions under which SAG1 (and to lesser extent SAG2A) would be able to form proper disulfide bonds in the reducing environment of *E. coli* and at the same time would result in soluble, biotinylated and N-terminal fusion-free proteins. MBP and GST are two widely used fusion partners supporting enhanced solubility and stability but also provide an affinity handle for purification. Both proteins were already used in the past as fusions with SAG1/SAG2A for diagnostic purposes [10–12, 21, 26, 47, 55, 56].

Here we provide direct quantitative evidence for a substantial influence of N-terminal fusion partners on the binding of human antibodies to SAG1. This is consistent with Graille et al. [30] who reported that the major epitope of SAG1 is at the N-terminus. Thus, MBP or GST protein tags could hinder antibody access. This notion is also supported by a recent BBMA study that included GST-SAG1 fusion protein bound with its N-terminus to beads and which reported sensitivity and specificity of less than 87% towards human sera [11].

GST fusions have previously been described as a general method for the directed coupling of antigens to user-modified Luminex beads [57]. For this, a cross-linked casein-glutathione adduct is custom-synthesized via a three-step chemical procedure before it can be coupled via EDC/NHS chemistry to carboxylated beads. GST fusion proteins then bind with sufficient affinity (K_d_ = 6.9 × 10^− 9^ mol/L) to the beads [57]. However, this system has several drawbacks: (i) it requires lengthy synthesis of the casein-glutathione adduct; (ii) antibodies against GST are present in human populations exposed to the helminth *Schistosoma sp*., from which this protein is derived from [58]. This limits its usefulness in endemic areas and requires an additional control bead region with GST alone for the assay; (iii) GST, in contrast to MBP, has four cysteines, which could form non-intended disulfide bonds with the fusion partner [59], thereby compromising proper disulfide formation of an antigen, like in the case of SAG1/SAG2A.

In contrast, the SAG1_bio_-His_6_ can directly be added to MagPlex®-Avidin beads that are now commercially available, or, as described here, by chemical coupling of commercially available streptavidin to regular magnetic MagPlex® beads. Alternatively, SAG1_bio_-His_6_ can be directly immobilized onto non-magnetic commercial LumAvidin beads. The Avi-His_6_ tag adds only a 4 kDa additional C-terminal ‘tail’ which is expected not to interfere with antibody recognition or being recognized by human sera. A further advantage of SAG1_bio_-His_6_ is the extremely tight interaction with Sav (K_d_ ≈ 10^− 14^ mol/L). This makes a single affinity purification on a metal chelate matrix sufficient as all impurities can be washed away under stringent washing conditions after the incubation of SAG1_bio_-His_6_ with Sav-coupled MagPlex® beads. In fact, metal chelate affinity chromatography is only used to remove superfluous free biotin that would otherwise compete with SAG1_bio_-His_6_ binding to Sav.

## Conclusions

We have described a sophisticated, yet straightforward *E. coli* expression system for the production of the recombinant antigens SAG1 and SAG2A of the protozoan parasite *T. gondii* in soluble, correctly folded and C-terminally biotinylated forms (SAG1_bio_-His_6_ and SAG2A_bio_-His_6_). The two proteins were shown to react specifically and with high sensitivity with human infection sera in a BBMA format, which is based on the oriented immobilization of the proteins on Sav-coated magnetic beads. Taking advantage of the possibility to separate the N-terminal fusion partner MBP from SAG1_bio_-His_6_ via TEV protease, we showed that such a fusion partner can negatively influence the accessibility of human antibodies to the major N-terminal epitope of SAG1. We propose that both proteins in this format are attractive replacements (either alone or in combination) for the previously described *T. gondii* antigens in multiplex assays intended for large-scale seroepidemiological studies. The general expression strategy described herein will also be useful for other antigens where oriented immobilization is key for recognition by antibodies or ligands.

## Methods

### Sequence and structural analyses

The pairwise sequence alignment of SAG1 (SRS29B; TGGT1_233460; see ToxoDB.org) and SAG2A (SRS34A; TGME49_271050) according to Needleman-Wunsch was performed with EMBOSS Needle [60]. PredGPI [61] was used for cleavage site predictions of GPI-anchored proteins. For homology modeling of SAG2A onto SAG1 (PDB 1KZQ) [17], the SWISS-MODEL server was used [62]. 3D structures were inspected and visualized with UCSF Chimera 1.11.2 [63], which was also used to calculate the solvent accessible surface area of SAG1’s lysines.

### Plasmid constructs

#### Construction of pAviTag-MBP-SAG1 and pAviTag-MBP-SAG2A

The coding sequence of SAG1 (aa 31–289) was PCR-amplified with Phusion polymerase (NEB Germany) using the primers 2CT-SAG1-a and 2CT-SAG1-s (see Additional file 7: Table S1 for primer sequences) from plasmid pSAG1-GPI [24] and inserted into SspI-cut p2CT-10 (a gift from Scott Gradia; Addgene plasmid # 55209) using the SLiCE (Seamless Ligation Cloning Extract) method [64]. The *E. coli* strain JM109 containing pKD56 [65] was used for extract preparation and for the expression of recombinant p2CT-MBP-SAG1, which encodes the entire mature SAG1 protein from *T. gondii* strain RH with a maltose binding protein (MBP) fusion separated by 10 asparagine residues, and followed by a TEV protease cleavage site (tev; see Fig. 3c). This plasmid served as the template to amplify MBP-SAG1 (without the N-terminal 6 histidines (His_6_)) with primers MBP-pAvi-fwd and SAG-pAvi-rev for cloning into pAviTag-C-Kan (Expresso Biotin Cloning and Expression System; Lucigen) following the supplier’s instructions. The resulting plasmid, pAviTag-MBP-SAG1, encoded the MBP-SAG1 fusion protein with a C-terminal biotinylation tag (AviTag), followed by His_6_ for purification of full-length proteins via metal chelate affinity chromatography upon induction with rhamnose (Fig. 3).

For cloning of SAG2A, the genomic DNA from strain RH was used as template for PCR amplification using Phusion polymerase with the primers MBP-SAG2A-fwd and SAG2A-Avi-rev. The resulting fragment encodes the sequence from amino acids 27 to 162 and was cloned via SLiCE into BamHI- and PstI-cut pAviTag-MBP-SAG1. This expression plasmid was called pAviTag-MBP-SAG2A (Fig. 3a, b).

#### Construction of pBAD1030G-TB and pBAD1030G-B

For co-expression of the TEV protease and biotin ligase (BirA), the fused genes (Additional file 4: Supplementary Figure S1B) were re-amplified from plasmid pCTAB (unpublished) where they had been previously assembled via circular polymerase extension cloning (CPEC) [66] using plasmids pRK793 [34] and pDW363 [67] as templates (both plasmids a gift from David Waugh (Addgene plasmid # 8827 and # 8842)). Using primers pRSF1030G-fwd/ -rev and Phusion polymerase for PCR amplification, the resulting product was cloned using SLiCE into the plasmid pBAD1030G [68] (a kind gift of John E. Cronan). The resulting plasmid pBAD1030G-TB allowed expression of TEV protease and BirA as two separate proteins upon self-cleavage of the fusion protein at the internal tev site (Fig. 3a; Additional file 4: Supplementary Figure S1B) [40]. To obtain a plasmid without TEV, pBAD1030G-TB was digested with NcoI and BspHI (which removes the TEV coding sequence and produces compatible overhangs) and then religated to yield plasmid pBAD1030G-B, expressing BirA only.

The sequences of all relevant parts of newly assembled plasmids were confirmed by Sanger sequencing. The sequences of the expression constructs except pMJS9 are provided as Additional files 2, 3, 5, 6: Sequence S1, S2, S3 and S4.

### *E. coli* strains BioSAG1 and BioSAG2A

We transformed plasmids pAviTag-MBP-SAG1 (or pAviTag-MBP-SAG2A), pBAD1030G-TB and pMJS9 (expressing the codon-optimized sulfhydryl oxidase Erv1p from *S. cerevisiae* and codon-optimized human protein disulfide isomerase (PDI) [36]) into *E. coli* SHuffle (NEB). The three plasmids possess different antibiotic resistance genes (for kanamycin, gentamycin and chloramphenicol, respectively) as well as compatible replication origins (Fig. 3a). This allowed their stable propagation in the resulting strains, which were named *BioSAG1* or *BioSAG2A*, respectively*.*

### Expression and purification of recombinant proteins

For expression of SAG1 or SAG2A, the *BioSAG1* or *BioSAG2A* strains were grown in 500 mL LB medium at 37 °C to an OD_600_ of 0.5–0.6. Then the expression was pre-induced by the addition of arabinose (final concentration 0.5%) for two hours at 37 °C before SAG1 or SAG2A induction by rhamnose (final concentration 0.2%). The medium was also supplemented with biotin (50 μM final concentration). Induced cultures were then incubated for 18 h at 30 °C before centrifugation and resuspension of the pellet in 10 mL lysis buffer (50 mM NaH_2_PO_4_, 300 mM NaCl, 10 mM imidazole; containing cOmplete EDTA-free protease inhibitors (Roche); 1000 U Benzonase and 1 mg/mL lysozyme), followed by 30 min incubation at 4 °C. Cell disruption was performed by ultrasonication. The cleared cell lysates were passed over a 1 mL HisTALON Superflow Cartridge (TaKaRa) for metal chelate affinity chromatography, with 50 mM NaH_2_PO_4_, 300 mM NaCl, 20 mM imidazole as wash buffer. The bound fractions were eluted by a linear imidazole gradient from 20 to 500 mM and the eluates were then pooled. The buffer was subsequently exchanged to PBS on a 5 mL HiTrap Desalting column (GE Healthcare). This column was directly attached to a 1 mL MBP-Trap HP column (GE Healthcare) for final removal of MBP. All chromatographic procedures were performed on an ÄktaPurifier FPLC system as described by the manufacturer (GE Healthcare). The protein concentration was determined using the BCA assay (Thermo Fisher).

### SDS-PAGE, Western blot analysis and antibodies

10% or 12% SDS-PAGE, silver staining and Western blotting were performed using standard protocols. Staining/destaining of nitrocellulose membranes with DirectBlue 71 was performed as described [69]. The following primary and secondary antibodies were used with the indicated dilutions: mouse anti-MBP monoclonal antibody (NEB) (1:1000); mouse anti-6His tag monoclonal antibody (MAK 1396; Linaris GmbH) (1:2000); mouse anti-BirA monoclonal antibody (5B11c3–3; Novus Biologicals) (1:1000); goat IgG anti-human IgG (Fc)-RPE (1:333); donkey anti-mouse IgG (H + L) RPE-F(ab’)_2_ fragment (1:500); streptavidin-HRPO (1:1000); goat IgG anti-mouse IgG (H + L)-HRPO (1:5000) (all Jackson ImmunoResearch Laboratories). The detection of secondary antibodies was performed using Super Signal West Dura Extended Duration Substrate (Pierce) according to the manufacturer’s instructions.

The description and evaluation of human sera used in this study as either seropositive or -negative using either the VIDAS TOXO IgG enzyme-linked fluorescent immunoassay (ELIFA; bioMérieux) or the anti-*Toxoplasma gondii*-IgG ELISA (Euroimmun, Lübeck, Germany) were published previously [14, 70].

### Streptavidin coupling to beads and sera analysis by BBMA

The coupling of recombinant Sav (Anaspec; 25 μg/1,5 × 10^6^ MagPlex® beads, region 33) and performing the BBMA proceeded according to the instructions of the xMAP® Cookbook [71] and have been described in detail previously [70]. We did not observe notable differences in binding of biotinylated antigens and concomitant maximal signal intensities with standard sera and different batches of custom-prepared Sav beads (data not shown). Depending on the preparation, between 10 and 100 ng SAG1_bio_-His_6_ (or SAG2A_bio_-His_6_) were added to 1500 Sav-coated beads (per well) and bound human antibodies were detected with a 1:333 dilution of goat IgG anti-human IgG (Fc)-RPE. Human serum albumin (Sigma-Aldrich) or unconjugated goat IgG anti-human IgG (H + L) (Jackson ImmunoResearch Laboratories) coupled to different bead regions were included as negative or positive controls, respectively [70].

### Data analysis

Data analyses (plotting, quantification, and statistical analyses) in Figs. 6 and 7 were performed using the open source statistics software R (version 3.5.1) [72] in conjunction with packages drLumi [73] and pROC [70, 74]. For other analyses Prism 8 (GraphPad) was used.

### In vitro TEV digestion and analysis of MBP_tev_-SAG1_bio_-His_6_F

For in vitro TEV digestion, 1.5 μg purified MBP_tev_-SAG1_bio_-His_6_ were first incubated either without or with 10 U TEV protease (NEB) in a final volume of 50 μl 1x TEV reaction buffer and incubated at 30 °C for 1 h or 4 h, or overnight at 4 °C. After incubation, 3 × 10^4^ Sav-coated MagPlex® beads were added, incubated with shaking for 1 h, then beads were washed and resuspended in PBS/1%BSA. A total of 1500 beads for each of the different conditions were then analyzed as above, using either the anti-MBP antibody followed by donkey anti-mouse-PE, or human sera followed by anti-human IgG (Fc)-RPE, as described above.

## Supplementary information

**Additional file 1.** **Movie S1.**

**Additional file 2. Sequence S1.**

**Additional file 3. Sequence S2.**

**Additional file 4. Supplementary Figure S1A.**

**Additional file 5. Sequence S3.**

**Additional file 6. Sequence S4.**

**Additional file 7. Table S1.**

**Additional file 8. Supplementary Fig. S2.**

## Data Availability

All data generated or analyzed during this study are included in this published article (and its supplementary information files). Plasmids are available upon request.

## Abbreviations

BBMA: Bead-based multiplex assay
BirA: Biotin ligase from *E. coli*
EDC: 1-Ethyl-3-[3- dimethylaminopropyl]carbodiimide hydrochloride
ELIFA: Enzyme-linked immunofluorescent assay
ELISA: Enzyme-linked immunosorbent assay
GPI: Glycosylphosphatidylinositol
GST: Glutathione-S-transferase
HRPO: Horseradish peroxidase
IU: International units
mab: Monoclonal antibody
MBP: Maltose-binding protein
MFI: Mean fluorescent intensity
PDI: Protein disulfide isomerase
R-PE: R-Phycoerythrin
SAS: Solvent accessible surface area
Sav: Streptavidin
SDS-PAG(E): Sodium dodecyl sulfate polyacrylamide gel (electrophoresis)
Sulfo-NHS: N-hydroxysulfosuccinimide
TEV: Tobacco Etch Virus
