# Supplementary material for: Expression of in vivo biotinylated recombinant antigens SAG1 and SAG2A from Toxoplasma gondii for improved seroepidemiological bead-based multiplex assays
Source: BMC Biotechnol. 2020 Oct 6;20:53. doi: 10.1186/s12896-020-00646-7 (PMC7542104; doi:10.1186/s12896-020-00646-7)
Supplement: Supplementary file 4 — Additional file 4. Supplementary Figure S1A. [file 12896_2020_646_MOESM4_ESM.pdf]

A

MGSSMKIEEGKLVIWINGDKGYNGLAEVGKKFEKDTGIKVTVEHPDKLEE
KFPQVAATGDGPDIIFWAHDRFGGYAQSGLLAEITPDKAFQDKLYPFTWD
AVRYNGKLIAYPIAVEALSLIYNKDLLPNPPKTWEEIPALDKELKAKGKS
ALMFNLQEPYFTWPLIAADGGYAFKYENGKYDIKDVGVDNAGAKAGLTFL
VDLIKNKHMNADTDYSIAEAAFNKGETAMTINGPWAWSNIDTSKVNYGVT
VLPTFKGQPSKPFVGVLSAGINAASPNKELAKEFLENYLLTDEGLEAVNK
DKPLGAVALKSYEEELAKDPRIAATMENAQKGEIMPNIPQMSAFWYAVRT
AVINAASGRQTVDEALKDAQTNSSSNNNNNNNNNNLGIEENLYFQ✂SDPP
LVANQVVTCPDKKSTAAVILTPTENHFTLKCPKTALTEPPTLAYSPNRQI
CPAGTTSSCTSKAVTLSSLIPEAEDSWWTGDSASLDTAGIKLTVPIEKFP
VTTQTFVVGCIKGDDAQSCMVTVTVQARASSVVNNVARCSYGADSTLGPV
KLSAEGPTTMTLVCGKDGVKVPQDNNQYCSGTTLTGCNEKSFKDILPKLT
ENPWQGNASSDKGATLTIKKEAFPAESKSVIIGCTGGSPEKHHCTVKLEF
AGAAGALSLSTPPTPSTPPTGLNDIFEAQKIEWHEHHHHHH*

Maltose-binding protein (MBP) 45 kDa

tev ✂ site

mSAG1 27 kDa

AviTag **& Biotin & 6His-Tag** 4 kDa

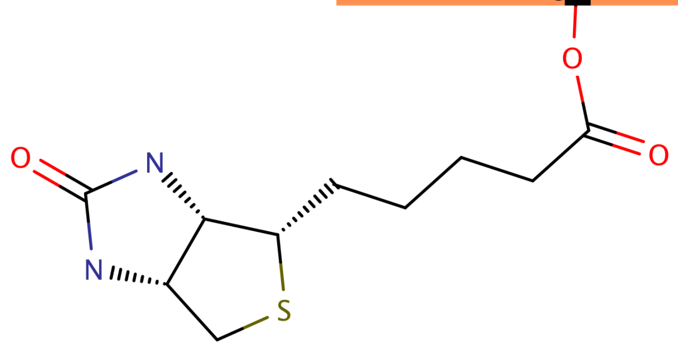

B

MGGESLFKGPRDYNPISSTICHLTNESDGHTTSLYGIGFGPFI
ITNKHLFRRNNGTLLVQSLHGVFKVKNTTTLQQHLIDGRDMIIIRMPKDF
PPFPQKLKFREPQREERICLVTTNFQTKSMSSMVSDTSCTFPSSDGIFWK
HWIQTKDGQCGSPLVSTRDGFIVGIHSASNFTNTNNYFTSVPKNFMELLT
NQEAQQWVSGWRLNADSVLWGGHKVFMVKPEEPFQPVKEATQLMNRRRRR
ENLYFQ✂GYPYDVPDYAMKDNTVPLKLIALLANGEFHSGEQLGETLGMSR
AAINKHIQTLRDWGVDVFTVPGKGYSLPEPIQLLNAEQILGQLDGGSVAV
LPVIDSTNQYLLDRIGELKSGDACVAEYQQAGRGRRGRKWFSPFGANLYL
SMFWRLEQGPAAAIGLSLVIGIVMAEVLRKLGADKVRVKWPNDLYLQDRK
LAGILVELTGKTGDAQIVIGAGINMAMRRVEESVVNQGWITLQEAGINL
DRNTLAAMLIRELRAALELFEQEGLAPYLSRWEKLDNFINRPVKLIIGDK
EIFGISRGIDKQGALLLEQDGIIKPWMGGEISLRSAEK*

TEV protease 29 kDa

tev ✂ site HA tag

Biotin ligase (BirA) 37 kDa

Fig. S1
