# Supplementary material for: Expression of in vivo biotinylated recombinant antigens SAG1 and SAG2A from Toxoplasma gondii for improved seroepidemiological bead-based multiplex assays
Source: BMC Biotechnol. 2020 Oct 6;20:53. doi: 10.1186/s12896-020-00646-7 (PMC7542104; doi:10.1186/s12896-020-00646-7)
Supplement: Supplementary file 7 — Additional file 7. Table S1. [file 12896_2020_646_MOESM7_ESM.pdf]

primer name	sequence (5' > 3')
2CT-SAG1-a	CGCGGATCCGTTATCCACTTCCAATTAGCTTATAGGGCCCCTGCAGCCCCGGCAAAC
2CT-SAG1-s	GGATCGAGGAAAACCTGTACTTCCAATCGGATCCCCCTCTTGTTG
MBP-pAvi-fwd	GAAGGAGATATACATATGGGTTCTTCTATGAAAATCGAA
SAG1-pAvi-rev	CGGCGGGGTGGATAAGCTTAGGGCCCCTGCAGCCCCGGC
pRSF1030G-fwd	GGGCTAGCAGGAGGAATTCACCATGGGAGAAAGCTTGTTTAAGGG
pRSF1030G-rev	CTCTAGAGGATCCCCGGGTACTTATTTTTCTGCACTACGCAGGG
MBP-SAG2A-fwd	GATCGAGGAAAACCTGTACTTCCAATCCACCACCGAGACGCCAGC
SAG2A-Avi-rev	TCGGCGGGGTGGATAAGCTTAGGGCCGTGAGAGACACAGGGTCAA

Table S1
