# Supplementary figures and images for: Expression of in vivo biotinylated recombinant antigens SAG1 and SAG2A from Toxoplasma gondii for improved seroepidemiological bead-based multiplex assays
Source: BMC Biotechnol. 2020 Oct 6;20:53. doi: 10.1186/s12896-020-00646-7 (PMC7542104; doi:10.1186/s12896-020-00646-7)

Fig. 4

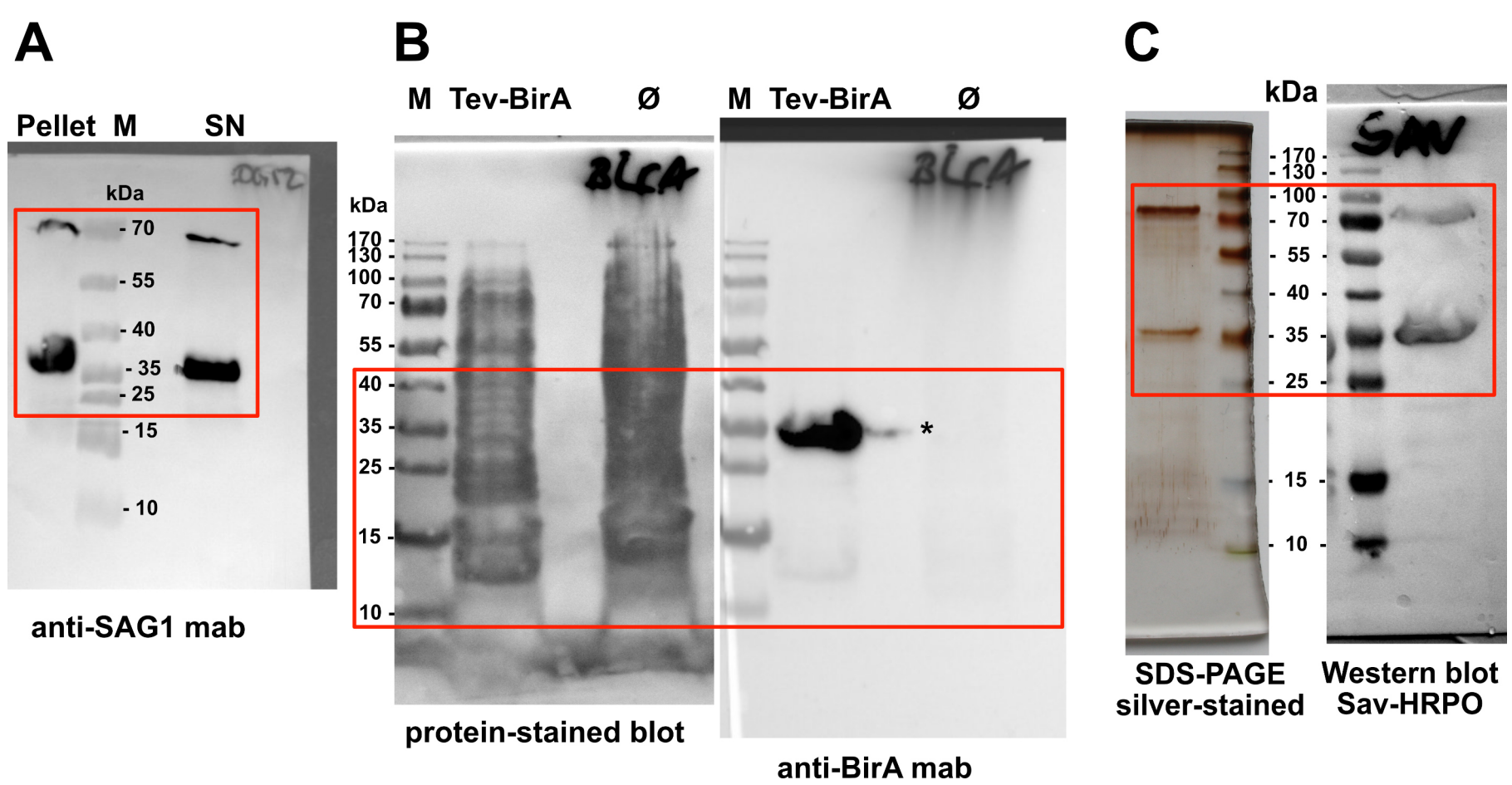

Fig. 5

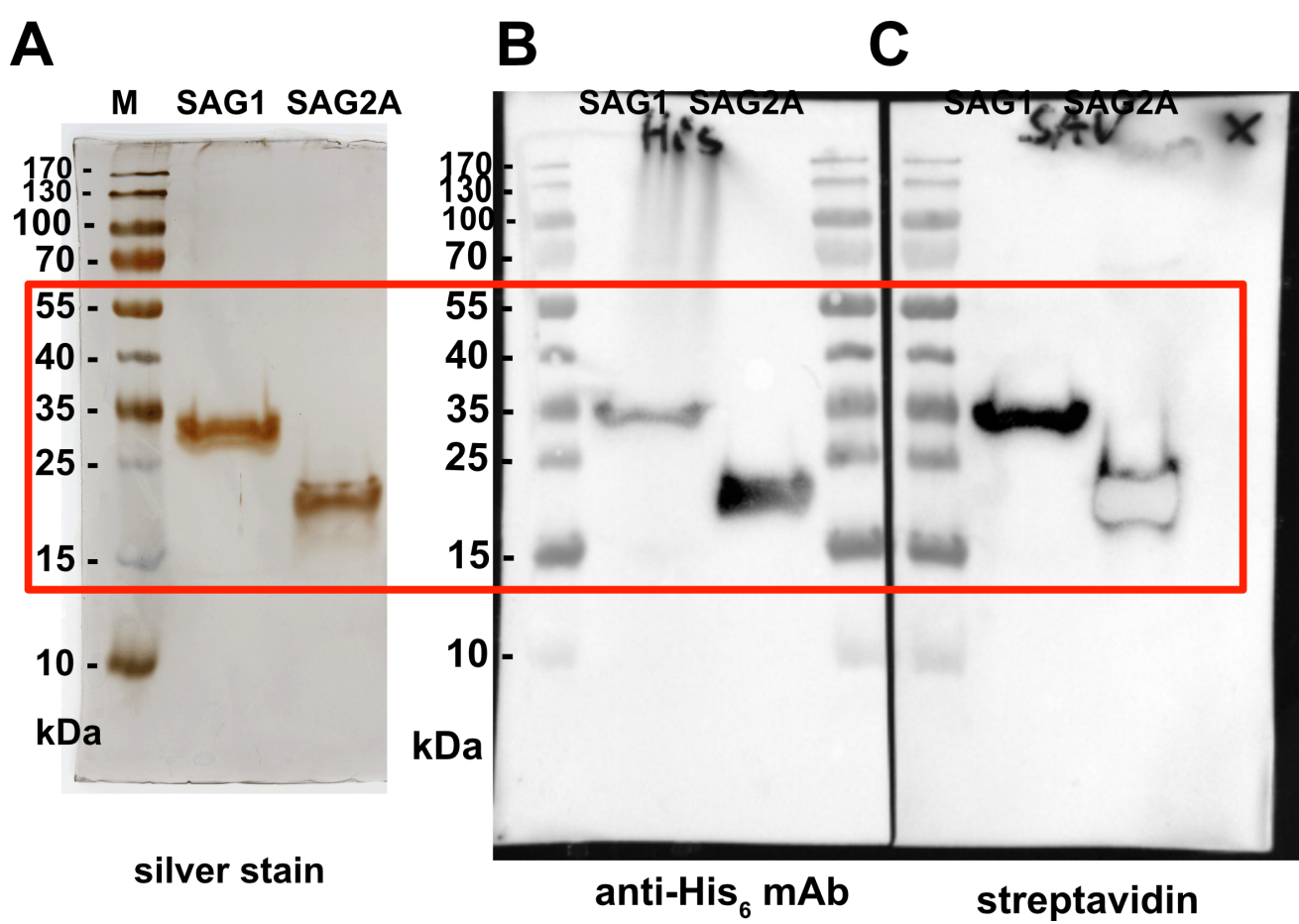

Fig. 8

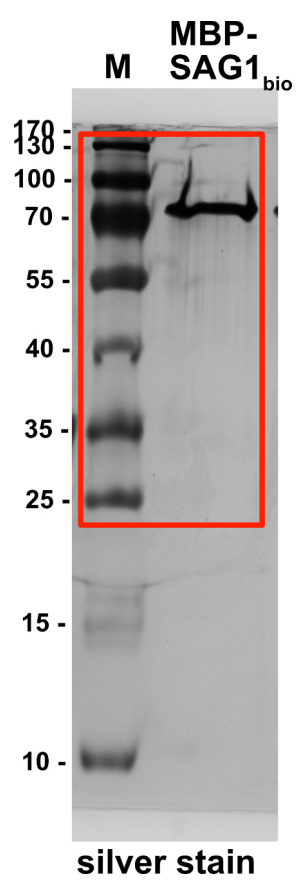

Supplement: Supplementary file 8 — Additional file 8. Supplementary Fig. S2. [file 12896_2020_646_MOESM8_ESM.pdf]
